# ROSes-FINDER: a multi-task deep learning framework for accurate prediction of microorganism reactive oxygen species scavenging enzymes

**DOI:** 10.3389/fmicb.2023.1245805

**Published:** 2023-09-07

**Authors:** Yueyang Yan, Zhanpeng Shi, Haijian Wei

**Affiliations:** ^1^College of Veterinary Medicine, Jilin University, Changchun, China; ^2^Department of Organ Transplantation, The Affiliated Yantai Yuhuangding Hospital of Qingdao University, Yantai City, China

**Keywords:** reactive oxygen species, multi-task deep learning, voting-based approach, oxidative stress, software engineering

## Abstract

Reactive oxygen species (ROS) are highly reactive molecules that play important roles in microbial biological processes. However, excessive accumulation of ROS can lead to oxidative stress and cellular damage. Microorganism have evolved a diverse suite of enzymes to mitigate the harmful effects of ROS. Accurate prediction of ROS scavenging enzymes classes (ROSes) is crucial for understanding the mechanisms of oxidative stress and developing strategies to combat related diseases. Nevertheless, the existing approaches for categorizing ROS-related proteins exhibit certain drawbacks with regards to their precision and inclusiveness. To address this, we propose a new multi-task deep learning framework called ROSes-FINDER. This framework integrates three component methods using a voting-based approach to predict multiple ROSes properties simultaneously. It can identify whether a given protein sequence is a ROSes and determine its type. The three component methods used in the framework are ROSes-CNN, which extracts raw sequence encoding features, ROSes-NN, which predicts protein functions based on sequence information, and ROSes-XGBoost, which performs functional classification using ensemble machine learning. Comprehensive experiments demonstrate the superior performance and robustness of our method. ROSes-FINDER is freely available at https://github.com/alienn233/ROSes-Finder for predicting ROSes classes.

## Introduction

Living organisms are constantly exposed to environmental stressors that can cause oxidative damage to their cells and tissues. To alleviate the ROS and induced antagonist effects, microbes rely on the well-evolved ROS-scavenging systems, which is comprised of enzymatic to balance the ROS levels at a steady-state (Johnson and Hug, 2019). ROS molecules, including superoxide anion, hydrogen peroxide, hydroxyl radical, and singlet oxygen, are produced due to normal cellular metabolism or exposure to environmental stressors (Borisov et al., 2021). While these ROS molecules play crucial roles in a variety of cellular processes, their excessive accumulation can lead to oxidative stress and cellular damage. Therefore, microbes have developed diverse defense mechanisms to combat ROS-induced damage, with ROSes playing important roles in scavenging ROS and protecting cells from oxidative damage (Salganik, 2001).

With the rapid advancement of high-throughput sequencing technologies, the volume of genomic and proteomic data generated has increased exponentially, leading to an explosion of sequencing data in recent years (Pai and Satpathy, 2021). While a multitude of bioinformatics tools and databases exist for protein annotation, such as DEEPARG and ARG_SHINE, these resources still have their own set of limitations (Bileschi et al., 2022), the identification of ROSes still poses a significant challenge (Ho Thanh Lam et al., 2020). This is due to the complex functional diversity of ROSes proteins and the lack of well-defined sequence motifs that are unique to these proteins. As a result, traditional protein annotation methods have become increasingly inefficient in keeping pace with the exponential growth of sequencing data (Chandra et al., 2023). This has created a pressing need for the development of fast and efficient computational methods for the accurate annotation of ROSes proteins, which can be accomplished through machine learning and deep learning techniques (Ko et al., 2020).

As high-throughput sequencing technologies continue to advance, the amount of protein sequence data is growing exponentially. However, the functions of most proteins still remain unclear (Vasina et al., 2022). Experimental and computational methods are the two major approaches to determining protein functions. While experimental methods rely on biological experiments to verify protein function, they are much slower than the speed of generating protein sequence data. In contrast, computational methods predict protein functions from protein sequence structures and other information, which is a more efficient and economical approach to determining protein function.

Sequence alignment-based methods, such as BLAST (Ye et al., 2006), are widely used for protein functional annotation. However, these methods have limitations in accurately predicting protein function based solely on sequence similarity. For instance, proteins with highly similar sequences may have different functions (Keskin and Nussinov, 2005), while proteins with low sequence similarity may perform similar functions (Bork and Koonin, 1998). Additionally, these methods may not be effective in identifying remote homologs or proteins with divergent sequences, leading to incorrect functional annotations. Moreover, sequence alignment-based methods do not consider other critical factors, such as protein structure (Kuhlman and Bradley, 2019), post-translational modifications, and protein–protein interactions (Ramazi and Zahiri, 2021), which can significantly influence protein function.

To overcome these limitations, alternative computational methods have been developed, such as machine learning-based approaches. These methods can integrate multiple sources of data, including sequence information, protein structure, and functional annotations from various databases, to improve the accuracy of protein function prediction. By leveraging these methods, we can accelerate the process of determining protein function and unlock the potential of the exponentially growing protein sequence data. This study proposes a new integrated approach called ROSes-FINDER for predicting anti-oxidative protein classes, which overcomes the limitations of existing methods for classifying ROSes. ROSes-FINDER is a multi-task deep learning framework that predicts multiple ROS properties simultaneously, including whether the input protein sequence is ROSes and, if so, what type of ROSes protein it belongs to. To improve the accuracy and robustness of the integrated model, the framework integrates three component methods using a voting-based approach, namely ROSes-CNN, ROSes-NN, and ROSes-XGBOOST. ROSes-CNN uses raw sequence encoding for feature extraction, while ROSes-NN is suitable for predicting protein functions based on sequence information by learning complex non-linear relationships. ROSes-XGBOOST is an ensemble machine learning algorithm that combines the outputs of many decision trees to make a final prediction, making it a useful tool for functional classification. The combination of these three methods can potentially provide more comprehensive and reliable functional predictions for proteins, and the voting-based approach reduces the impact of individual classifier errors, thereby reducing the risk of overfitting.

## Materials and methods

In this section, we present (i) a description of the benchmark dataset, (ii) overview of ROS_Finder, (iii) the implementation of three proposed component methods used for integration and the ensemble model, and (iv) implementation details.

### Database description

**Figure 1:**
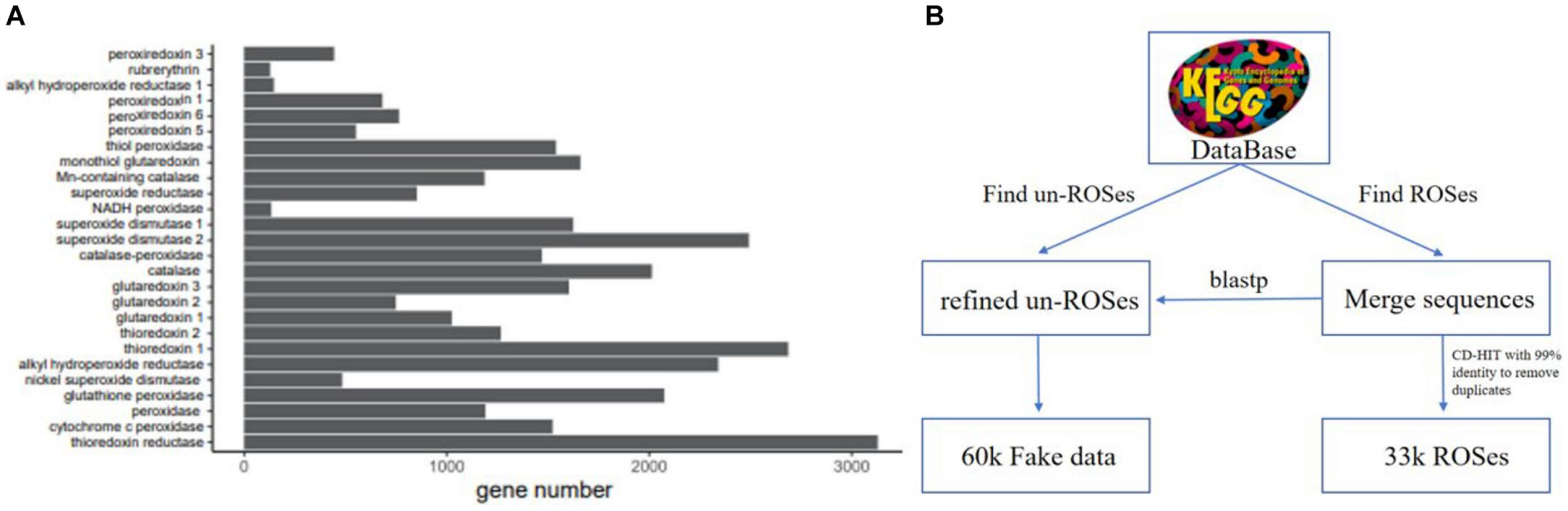
ROSes database composition and the ROSes database construction pipeline. **(A)** The statistics of the ROSes database. The number of sequences belonging to each ROSes family is different. **(B)** To construct the database, we merged the sequences from KEGG databases, followed by a post-processing step to remove duplicates. Then, we utilized scripts to automatically gather relevant information about ROSes proteins from the KEGG database and generate labels for sequences. Subsequently, we engaged in manual curation, classifying each sample into ROSes families and clearly delineating their functions. Aspects to each sequence in the database.

We have developed a multi-label ROS database called ROS-DB (Figure 1), based on the Kyoto Encyclopedia of Genes and Genomes (KEGG) (Kanehisa et al., 2021a), which has been highly curated and confidently annotated. We chose to use the KEGG database annotations, as they allow for more precise protein annotations and greater confidence in protein annotation than databases such as Pfam (Kanehisa et al., 2021b). The first branch serves a dual purpose: it identifies ROS-positive samples and gathers a substantial number of unfiltered ROS-negative samples. Following this, the subsequent second branch comes into play. In this step, the unfiltered ROS-negative samples are subjected to a blastp sequence comparison against the ROS-positive samples. This comparison yields a refined set of 59,893 non-ROSes sequences from databases. These sequences exhibit the highest BLAST similarity scores with the ARGs in the ROSes-DB. This refined set is then utilized as negative sets, deliberately designed to resemble the positive set as closely as possible. By employing this strategy, we compel ROS_FINDER to develop a more robust and potent model.

To construct the KEGG-ROS-99 dataset, we performed CD-HIT (Mistry et al., 2021) clustering on the KEGG dataset with a 99% identity threshold, removing identical and redundant sequences to obtain sequences with higher identity scores. The representative sequences of the clusters generated by CD-HIT were then retained in the fasta file. Next, we labeled these sequences from three perspectives and manually checked them for (1) the category of ROS scavenging enzymes to which they belong and (2) the mechanism of ROS scavenging enzymes.

The resulting ROS-DB database consists of 33,748 high-quality sequences, each labeled with one of 26 ROS scavenging enzyme categories. This type of multi-class database ensures that the trained model can automatically capture the most relevant features associated with ROS scavenging enzymes. This type of multi-class database ensures that the trained model can automatically capture the most relevant features associated with ROS scavenging enzymes. Over 9% of the genes belong to the thioredoxin reductase, and approximately 24% of the genes were assigned to the cytochrome c peroxidase.

### Overview of ROS-Finder

**Figure 2:**
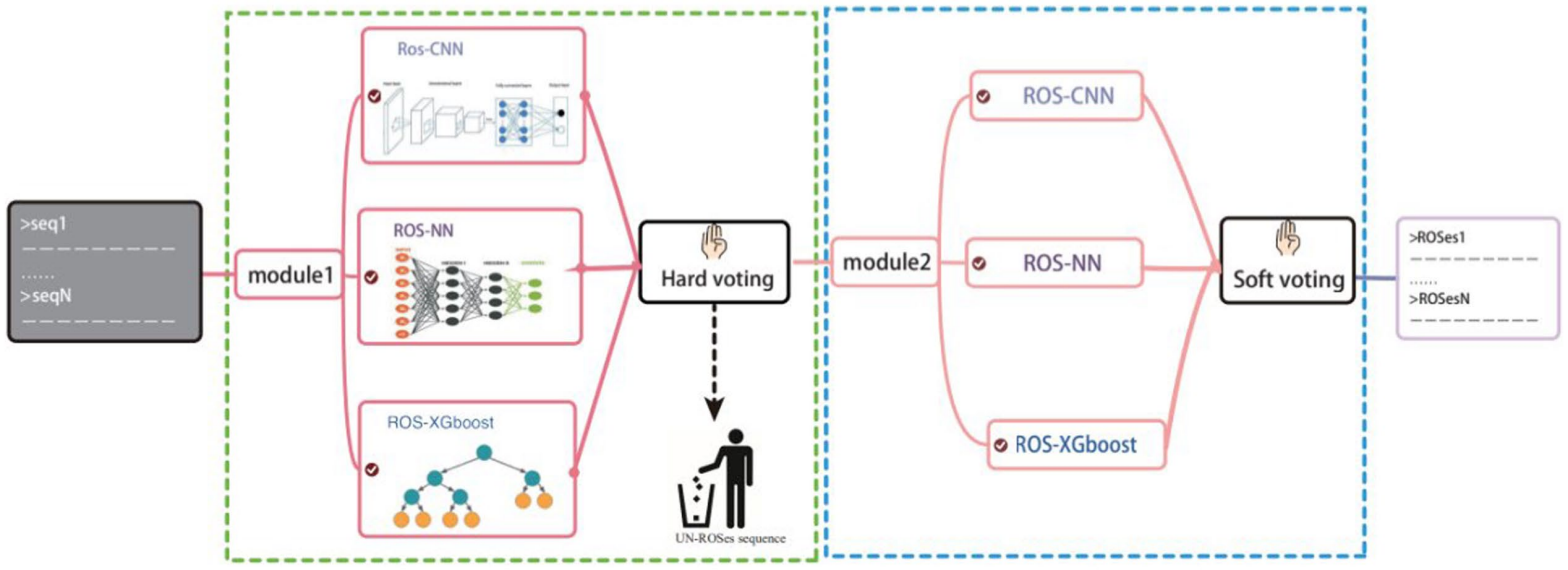
Illustrates the ROSes-Finder framework, which consists of two levels: (i) the “component module,” which obtains prediction results based on three different algorithms and different input information: ROSes-CNN (natural language learning), ROSes-ANN (protein sequence information), and ROSes-XGBoost (The composition of k-spaced acid pairs, CKSAAP); and (ii) the “integration module,” which uses a voting algorithm to generate predictions from the “component module” and improve overall performance.

ROS-Finder is a supervised machine learning framework specifically designed for identifying ROS scavenger enzymes through analyzing annotations in the ROS annotation space (Figure 2). The framework adopts a hierarchical prediction strategy that deploys a layered structure for ROS scavenger enzyme classification. Given a protein sequence, ROS_Finder first classifies it as a ROS scavenger enzyme or non-ROS scavenger enzyme. If the input sequence is a ROS scavenger enzyme, we predict which ROS scavenger enzyme category it belongs to. Therefore, for any sequence analyzed by the ROS_Finder framework, the first model (level 1) predicts whether it is a ROS scavenger enzyme or non-ROS scavenger enzyme. If it is a ROS scavenger enzyme, the second model (level 2) predicts its ROS scavenger enzyme category and molecular sub-class.

### Component module: three different component methods using different features (ROSes-CNN, ROSes-NN, and ROSes-XGBoost)

#### ROSes-CNN

Convolutional neural networks (CNN) play a crucial role in protein function prediction by analyzing protein sequences and identifying patterns and features associated with their functions (Kattenborn et al., 2021).

For these models, the input is a protein sequence, which is a string of 23 characters representing different amino acids. To prepare the input for deep learning mathematical models, preprocessing is done first. The protein sequences are tokenized into numbers using the Tokenizer function from the Keras (Kathuria et al., 2019). This function first calculates the frequency of each character across all sequences, and then maps the top N characters with the highest frequency to the numbers 1 to N, the next N characters with the next highest frequency to the numbers N + 1 to 2 N, and so on. The numerical sequences are then padded to a fixed length using the pad_sequences function so that the neural network can process them (Jang et al., 2019). Through this preprocessing process, the protein sequence data and label data are converted into PyTorch tensors, so that they can be used as input for training the neural network model.

We used a convolutional neural network (CNN) with an attention mechanism for the text classification task. The model is implemented using PyTorch. We defined a CNN-based text classification model that includes an embedding layer, three convolutional layers, a linear layer, and a self-attention layer. The embedding layer embeds the input text sequence into a low-dimensional vector space, the convolutional layers and the linear layer are used for feature extraction and classification, and the self-attention layer is used for extracting important information from the text sequence. We then defined the hyperparameters and optimizer required for training. The optimizer uses the Adam optimization algorithm, and the cross-entropy loss function is used to calculate the difference between the model’s predicted results and the true labels. This loss function encourages the model to assign higher probabilities to the correct class while penalizing incorrect predictions. Finally, we loaded the training and testing datasets and started training the model.

#### ROSes-NN

CKSAAGP is a feature descriptor used in bioinformatics to represent protein sequences (Manavalan and Patra, 2022). The acronym stands for “Composition of k-spaced Amino Acid Pairs with Gap Penalty.” The descriptor takes into account the amino acid composition and the k-spaced pairs of amino acids in a sequence, with a penalty for gaps between them. We calculated the CKSAAGP as a protein chemical feature for each sequence. This module is based on neural networks (NN) for classification (Li et al., 2022). NN contains four linear layers with input and output dimensions of 20, 16, 8, 4, and 2, respectively. The forward method defines the process of forward propagation and uses the ReLU activation function. ReLU has shown to be effective in handling the vanishing gradient problem and accelerating convergence during training. The introduction of this non-linear activation function can make the neural network have stronger expression ability, thereby improving its performance. In the training process, the SGD optimizer (Stochastic Gradient Descent) plays a crucial role in machine learning and deep learning. It operates by iteratively adjusting the model’s parameters to minimize the loss function. This is achieved by moving in the direction of the steepest decrease, utilizing a subset of training data in each step. The CrossEntropyLoss loss function is employed to quantify the dissimilarity between predicted class probabilities and the actual target labels. It’s particularly effective for multi-class classification tasks like protein categorization. By encouraging the model to assign high probabilities to the correct class while penalizing incorrect predictions, it aids in enhancing the accuracy of the network’s classifications. Within the mentioned training method, the combination of the SGD optimizer and the CrossEntropyLoss loss function works harmoniously to fine-tune the model’s parameters, leading to precise protein classification. The model’s efficacy is then assessed by evaluating its performance on a separate test dataset.

#### ROSes-XGBoost

XGBoost stands for “Extreme Gradient Boosting,” which is a powerful and efficient machine learning algorithm used for supervised learning tasks, especially in classification and regression problems (Sagi and Rokach, 2021). The core idea behind XGBoost is to combine the predictions of several weak models, called decision trees, into a single strong model. In each iteration, XGBoost trains a new decision tree to fit the residuals of the previous iteration (Ma et al., 2021). The prediction of each tree is added to the overall prediction, and the algorithm continues to iterate until the loss function reaches a minimum or a user-specified stopping criteria is met. To improve the performance and prevent overfitting, XGBoost includes several regularization techniques, such as L1 and L2 regularization, tree pruning, and early stopping (Qiu et al., 2022). To train the XGBoost model, we calculated protein chemical features for each sequence and used them as inputs. We utilized specific parameter values in our XGBoost model as outlined: Learning Rate (0.1), max_depth (20), n_estimators (150), Gamma (0), Subsample (0.9). These choices were based on preliminary experimentation and were tailored to optimize the model’s performance on our dataset.

### Ensemble model voting algorithm

The voting algorithm is a type of ensemble learning method that combines the predictions of multiple classifiers to produce more accurate predictions (Qiu et al., 2022). In the ROSes-FINDER framework, we have incorporated a strategic implementation of three distinct algorithms. This deliberate inclusion of multiple algorithms holds the potential to enhance the overall robustness of the model. The rationale behind this lies in the prospect that variations in predictions stemming from different algorithms could serve to counterbalance potential errors. This, in turn, contributes to the reduction of the overall error rate, ultimately bolstering the model’s accuracy and reliability.

In more detail, the ensemble nature of our approach involves the deployment of these three algorithms. Initially, the first algorithm generates a preliminary prediction. This preliminary prediction forms a critical foundation, as it serves as a basis for further analysis. Subsequently, a collective decision is made through a hard voting mechanism, which takes into account the predictions of all three algorithms. This collective decision is pivotal in determining whether the given instance can be classified as a ROSes or not. Once a consensus is reached that a given instance indeed belongs to the ROSes class, a more nuanced approach comes into play. This involves employing a soft voting strategy to discern the specific class within the ROSes category. This finer classification process leverages the combined strength of all three algorithms, culminating in a more refined and accurate classification outcome.

### Implementation details

We collected 60 k non-ROSes from databases, which have the highest BLAST similarity scores to the ARGs in ROSes-DB, and used them as negative sets so that they resemble the positive set as much as possible, forcing ROS_FINDER to learn a more powerful model. Subsequently, we trained the 0-level model using KEGG data. During model training, we first converted each protein into three feature matrices. A single feature may be affected by noise, missing data, and other factors, leading to a decrease in classifier performance. Using multiple features can make the model more robust because the interactions between different features can provide more information, reducing the impact of specific features on classification.

Within the ROSes-FINDER framework, we set three different algorithms. The differences between different algorithms can help improve the robustness of the model because errors from different algorithms may cancel each other out, reducing the overall error rate. The first model first predicts based on the three algorithms, and then determines whether it is a ROSes through hard voting. If it is determined to be a ROSes, then soft voting is used to determine its class.

## Results

### Overall performance of ROSes-FINDER

**Figure 3:**
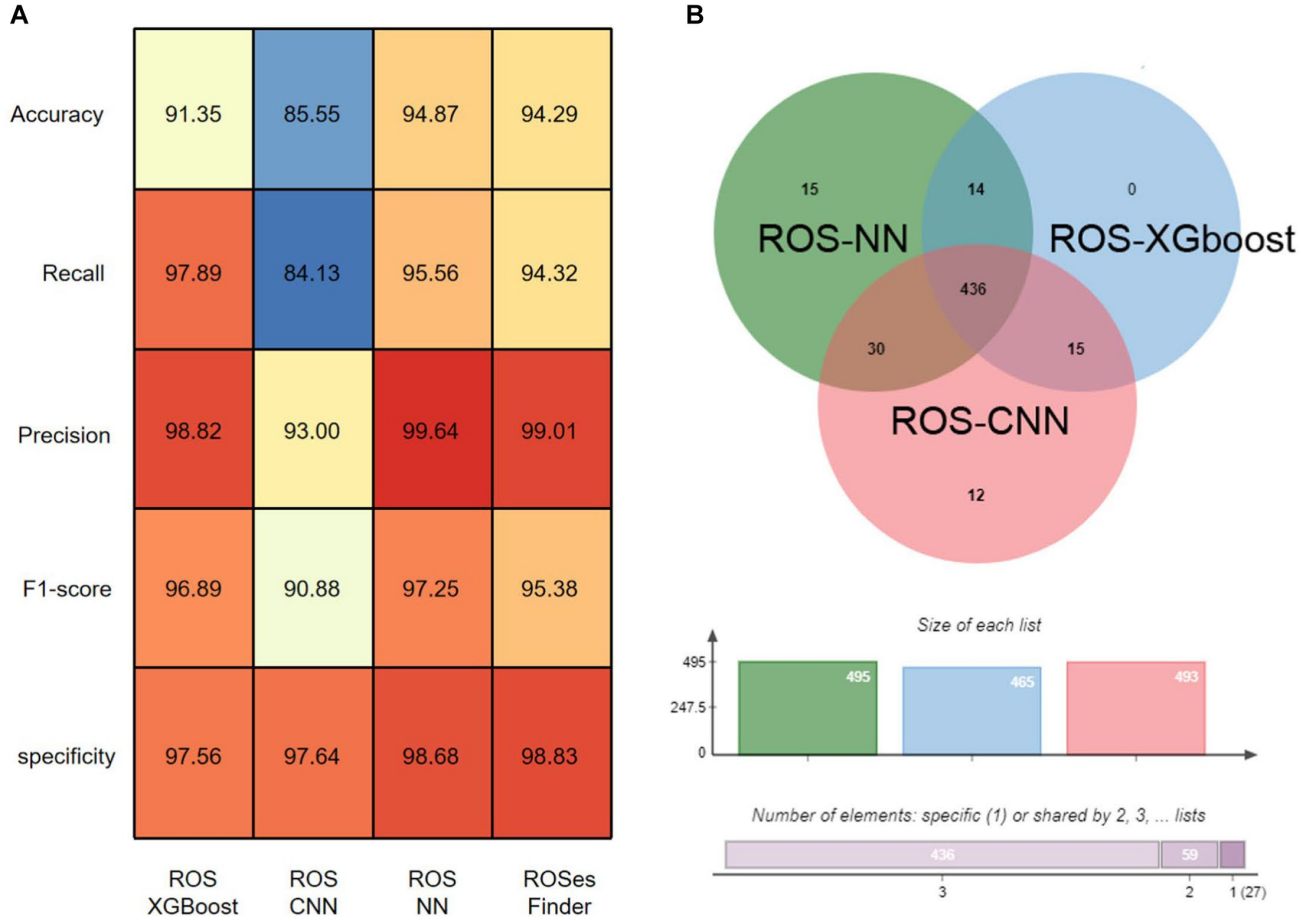
Overall performance of ROSes-Finder. **(A)** Shows the accuracy, recall, sensitivity, and F1 score of various methods on the test data. **(B)** Displays the Venn diagram of sequences correctly classified by the three-component method in the test data.

Based on our experimental results, we compared three classification algorithms (Figure 3A), namely ROSes-NN, ROSes-CNN, and ROSes-XGBoost, and obtained the results using a hard voting algorithm. We measured the accuracy, recall, precision, sensitivity, and F1 score of each algorithm on the test set for the classification task. Among the independent methods, ROSes-NN performed the best in terms of accuracy, recall, specificity and F1 score, but its precision was lower than that of ROSes-XGBoost. ROSes-XGBoost had relatively higher recall, but lower accuracy and F1 score. ROS-CNN had the highest precision, but its performance in other metrics was relatively poor. The results obtained by the hard voting algorithm (hard) were better than those of CNN in terms of accuracy, recall, sensitivity, and F1 score, but slightly lower than the best-performing algorithm ROSes-NN, with the highest precision. As for the ensemble model voting algorithm, the hard voting algorithm had better results in terms of accuracy, recall, sensitivity, and F1 score than the single algorithm ROSes-CNN, but slightly lower than the best-performing algorithm ROSes-NN. This indicates that integrating the prediction results of multiple algorithms through the hard voting algorithm can improve the performance of the model, especially when some algorithms perform poorly. The hard voting algorithm can reduce misclassification rate and overfitting risk by synthesizing the prediction results of multiple algorithms, thereby improving the robustness and generalization ability of the model.

### Effective expansion of protein discovery scope through integrative analysis using three methods

Next, we investigated the contribution of each component method to the overall performance of ROS_Finder. Among all 1,703 test sequences, 15 and 12 sequences were uniquely correctly classified by ROS-NN and ROS-CNN, respectively, indicating their outstanding performance in identifying these particular sequences (Figure 3B). A total of 87.6% of the sequences were correctly classified by all three component methods, reflecting the complementarity and superior integrative effect of ROSes-NN, ROSes-CNN, and ROSes-XGBoost. Specifically, we noted that ROSes-NN had the most outstanding performance in providing uniquely correct classification sequences. Among all test sequences, ROSes-NN provided 498 uniquely correct classification sequences for ROSes-Finder.

### The predictive performance for each ROSes class

**Figure 4:**
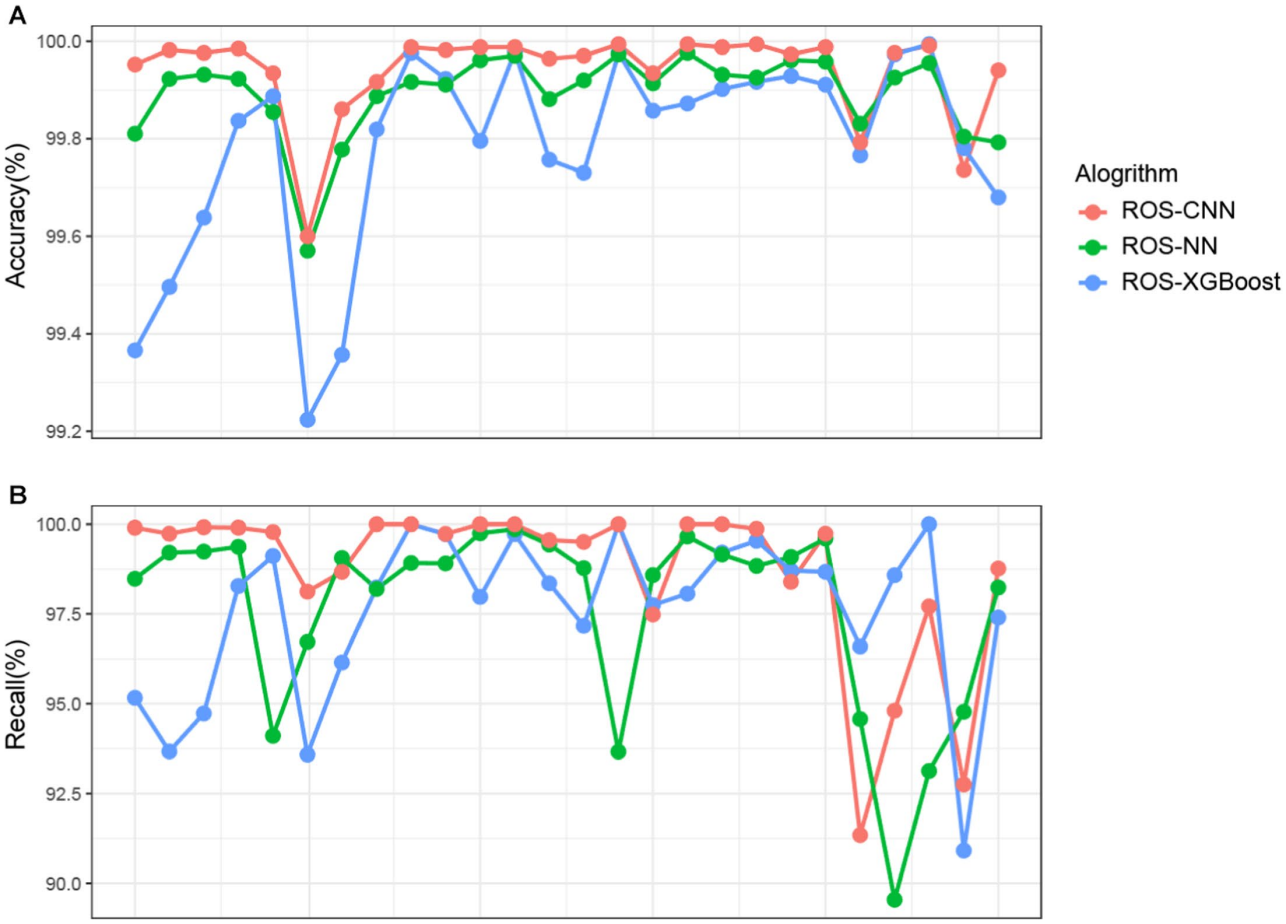
Detailed comparison of prediction performance. Each row represents a category. Each point represents a class for a particular algorithm. (Red points for ROS-CNN, green points for ROS-NN, blue points for ROS-XGBoost). Each column represents a category.

We next present three algorithms, ROSes-NN, ROSes-CNN, and ROSes-XGBoost, and their accuracy and recall in classifying different categories. All three algorithms demonstrate high accuracy (Figure 4A) in classifying different categories. In particular, the ROS-CNN algorithm performs better than the other two algorithms in most categories. To further evaluate the performance of these algorithms, we calculated their recall (Figure 4B). Recall is an indicator of the classification model’s performance, which represents the proportion of samples that belong to a certain category that the model correctly predicts. It can be observed that there is a significant difference in performance for different categories under different algorithms. For example, Category 4 performs well under the XGBoost algorithm but poorly under the NN algorithm, while Category 22 performs well under both the XGBoost and NN algorithms but poorly under the NN algorithm. Some categories have relatively low recall rates for at least one algorithm, suggesting that combining different algorithms may result in better overall results for different categories.

### Validation on novel ROSes

To validate the performance of our model in predicting ROSes, we selected four sequences from bacteria (Wang et al., 2020), which have been experimentally verified. Among them, one sequences had lower identity scores (<50%, BLAST) compared to our ROSes database training set. ROSes-Finder successfully predicted all sequences as ROSes and classified them correctly. This indicates that the ROSes-Finder model can accurately predict ROSes sequences from different types of organisms, even when these sequences have lower similarity to the ones in the training set. This also suggests that the ROSes-Finder model remains effective in handling sequences with certain degrees of variability, which is essential in practical applications. These results demonstrate the reliable performance of the ROSes-Finder model in predicting ROSes.

## Discussion

ROSes-FINDER is a hierarchical multi-task deep learning algorithm designed to predict microorganisms ROSes protein classes. Protein function annotation is a crucial process in bioinformatics, which helps in understanding the functions of proteins and their roles in biological processes (Chen et al., 2020). With the increase in the number of available protein sequences, it has become challenging to annotate protein functions manually. Hence, computational methods have been developed to automate the annotation ROSes process. This methods utilize machine learning algorithms and deep learning frameworks to predict and classify ROSes properties based on protein sequence information. Such automated annotation tools have the potential to accelerate the pace of research in this field.

The use of deep learning techniques, including convolutional neural networks (CNN), artificial neural networks (NN), and XGBoost algorithms, has been gaining traction in recent years for protein function prediction (Ejigu and Jung, 2020). These methods are particularly well-suited for protein function annotation because they can capture complex patterns and relationships between protein sequences and their functions. ROSes classification is a multi-class prediction problem, and ROSes-FINDER uses a multi-task learning approach to simultaneously predict multiple ROS properties. The use of a hierarchical model in ROSes-FINDER allows the model to learn high-level representations of the input data, which enables it to make accurate predictions even on proteins with limited sequence similarity. The hierarchical model used in ROSes-FINDER allows the model to learn representations of the input data at different levels of abstraction, resulting in a more comprehensive understanding of protein function.

The integration of multiple component methods is a key aspect of the ROSes-FINDER framework. This includes the use of convolutional neural networks (CNN), artificial neural networks (NN), and XGBoost algorithms, which each have unique strengths in analyzing protein sequence data. By combining these methods, ROSes-FINDER is able to achieve a higher level of accuracy and robustness in predicting multiple ROS properties simultaneously. The CNN component of ROSes-FINDER is used for raw sequence encoding feature extraction, which is particularly effective in capturing spatial information and patterns within the protein sequence. The NN component utilizes sequence information to predict protein function, while the XGBoost component is used for functional classification using ensemble machine learning. Through the integration of these methods, ROSes-FINDER is able to provide a more comprehensive analysis of protein function than any single method alone.

ROSes-FINDER has some limitations that need to be considered. Firstly, it has limitations in handling raw reads, which may affect its prediction accuracy in certain cases. Secondly, it requires a significant amount of computational resources, which can be a challenge for researchers with limited access to high-performance computing. Lastly, its prediction results are affected by the length of input sequences, and it also has limited utilization of non-sequence information. Despite these limitations, ROSes-FINDER remains an effective deep learning algorithm for ROSes classification. However, careful evaluation of these limitations is necessary when applying the method and appropriate measures may need to be taken to enhance its prediction accuracy and expand its scope of application.

Overall, we believe that ROSes-Finder has the potential to become a valuable tool for mining ROSes. We will continue to improve and develop our framework to better serve the needs of the scientific communities.

## Data availability statement

The original contributions presented in the study are included in the article/Supplementary Material, further inquiries can be directed to the corresponding authors.

## Author contributions

HW designing research studies, conducting experiments, acquiring data, analyzing data, and writing – original draft. YY acquiring data, analyzing data, and writing – review and editing. ZS collecting samples and writing – review. All authors contributed to the article and approved the submitted version.

## Funding

HW was supported by Shandong Provincial Medical and Health Science and Technology Development Plan (Fund code: 202004050936). ZS was supported by College Student Innovation and Entrepreneurship Training Program (Fund code: 202310183325).

## Conflict of interest

The authors declare that the research was conducted in the absence of any commercial or financial relationships that could be construed as a potential conflict of interest.

## Publisher’s note

All claims expressed in this article are solely those of the authors and do not necessarily represent those of their affiliated organizations, or those of the publisher, the editors and the reviewers. Any product that may be evaluated in this article, or claim that may be made by its manufacturer, is not guaranteed or endorsed by the publisher.
